# Migration of Wintering Grey Plover From Southeast Asia to North‐Central Siberia Challenges Breeding Population Delineations in Russia

**DOI:** 10.1002/ece3.70815

**Published:** 2025-02-24

**Authors:** David Li, Max De Yuan Khoo, Richard B. Lanctot, Pavel S. Tomkovich, Zhijun Ma, Jun Rui Chow, Malcolm Chu Keong Soh, Shufen Yang, Choon Beng How, Adrian Loo, Robert Teo, Liang Jim Lim, Chee Chiew Leong, Kenneth Boon Hwee Er

**Affiliations:** ^1^ National Parks Board Singapore; ^2^ Migratory Bird Management Division U.S. Fish and Wildlife Service Anchorage Alaska USA; ^3^ Zoological Museum, Lomonosov Moscow State University Moscow Russia; ^4^ Ministry of Education Key Laboratory for Biodiversity Science and Ecological Engineering, National Observations and Research Station for Wetland Ecosystems of the Yangtze Estuary, Institute of Eco‐Chongming, School of Life Sciences Fudan University Shanghai China

## Abstract

Shorebird populations are declining across the world due to factors such as habitat loss and climate change. Identification of shorebird migration routes and important stopover sites can facilitate the implementation of strategic and effective conservation measures. Using a satellite transmitter, we successfully tracked the migration of one Grey Plover ( *Pluvialis squatarola* ) from its wintering grounds in Singapore north along the East Asian‐Australasian Flyway (EAAF) to its breeding grounds located east of the Taymyr Peninsula in north‐central Siberia. This provides the first evidence that the Singapore plover could be part of the Yamal/Taymyr population that is known to only migrate south via the East Atlantic Flyway and winter in Western Europe. After breeding, the bird took an unexpected westward migration towards northwestern Taymyr Peninsula where it stopped at two locations for 9 and 5 days, respectively, before migrating south through Central Asia. Prior to crossing the Himalayan mountains, however, the plover migrated east again from the Xinjiang Autonomous Region in northwest China to Jiangsu Province along the Yellow Sea, before turning south again to migrate along the EAAF to return to its wintering ground in Singapore. The plover's westward post‐breeding migration was contrary to prevailing winds, while the eastward migration north of the Himalayas was facilitated by strong easterly winds. The plover's westward migration post‐breeding may be attributed to acquiring additional food resources prior to its southward migration, and/or because it was searching for future breeding or staging grounds. Both possibilities may be associated with habitat changes occurring on their breeding grounds due to climate change. Further studies on the Grey Plovers wintering in Southeast Asia are needed to understand whether the migration route taken by this individual is representative of the species.

## Graphical abstract

Using satellite transmitter, we successfully tracked the migration of one Grey Plover ( *Pluvialis squatarola* ) from its wintering grounds in Singapore north along the East Asian‐Australasian Flyway (EAAF) to its breeding grounds located east of the Taymyr Peninsula in north‐central Siberia. After breeding, the bird took an unexpected westward migration towards northwestern Taymyr Peninsula before migrating south through Central Asia. Prior to crossing the Himalayan mountains, however, the plover migrated east again from the Xinjiang Autonomous Region in northwest China to Jiangsu Province along the Yellow Sea, before turning south again to migrate along the EAAF to return to its wintering ground in Singapore. The plover's westward post‐breeding migration was contrary to prevailing winds, while the eastward migration north of the Himalayas was facilitated by strong easterly winds. The plover's westward migration post‐breeding may be attributed to acquiring additional food resources prior to its southward migration, and/or because it was searching for future breeding or staging grounds. Both possibilities may be associated with habitat changes occurring on their breeding grounds due to climate change.

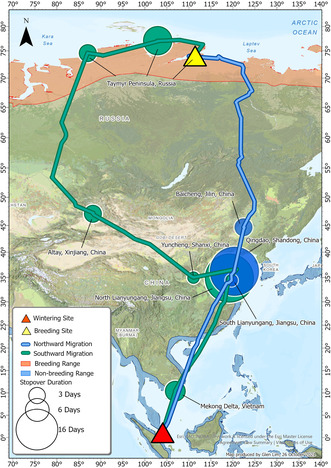

## 1 Introduction

Shorebird populations are declining globally primarily due to climate change and habitat loss (Kubelka et al. 2018; Santos et al. 2023). Arctic‐breeding shorebirds are increasingly challenged by changing climatic conditions on their breeding grounds (Meltofte 2013). These changes include phenological mismatches between the timing of reproduction and snowmelt, and hatching of chicks and invertebrate emergence/abundance (McKinnon et al. 2012; Martin and Wiebe 2004); range expansion or contraction as climatically suitable breeding conditions change with progressive warming (Wauchope et al. 2017; Taylor, Lanctot, and Holmes 2018); increasing numbers or changes in the predator community (Kubelka et al. 2018; Ims et al. 2019); and the arrival of new parasites (Kutz et al. 2005). This is further compounded by the migratory life‐history of shorebirds, which have suffered serious habitat loss and degradation in migration stopover and nonbreeding grounds (Piersma et al. 2016; Studds et al. 2017; Santos et al. 2023). Over half of the shorebird species breeding in the Arctic are exhibiting population declines (51% of 91 taxa with known trends) (Smith et al. 2020). Within the East Asian‐Australasian Flyway (EAAF), an estimated 88% of the shorebird species with known trends are in decline (15/17 taxa) (Smith et al. 2020). This has been attributed to the extensive loss of intertidal mudflats in East Asia, especially in the Yellow Sea (Studds et al. 2017; Murray et al. 2019). Based on demographic data collected by the Arctic Shorebird Demographic Network, factors at stopover or wintering sites rather than the breeding sites seemed to have a greater effect on the survival of adult shorebirds (Weiser et al. 2018, 2020). This finding indicates the importance of understanding where and how shorebirds migrate, especially in the EAAF so as to better understand the factors that may be limiting these populations.

Although bird ringing has been conducted in the EAAF since the early 1960s (McClure 1974; Minton et al. 2006; Bamford et al. 2008; Higgins and Davies 1996; Zhang and Yang 1997; Gan, Tan, and Li 2012; Wells 1999; Lagassé et al. 2020), information on the migratory routes of many arctic‐breeding shorebird species remains poorly known due to the limited number of re‐sightings and/or recoveries of ringed birds. Over the past few decades, the deployment of miniaturised tracking devices (e.g., geolocators and satellite transmitters) has revealed amazing migration routes and unknown stopover sites (e.g., Gill Jr. et al. 2009; Minton et al. 2011; Chan et al. 2019; Li et al. 2020). However, information is still lacking for many species.

**FIGURE 1:**
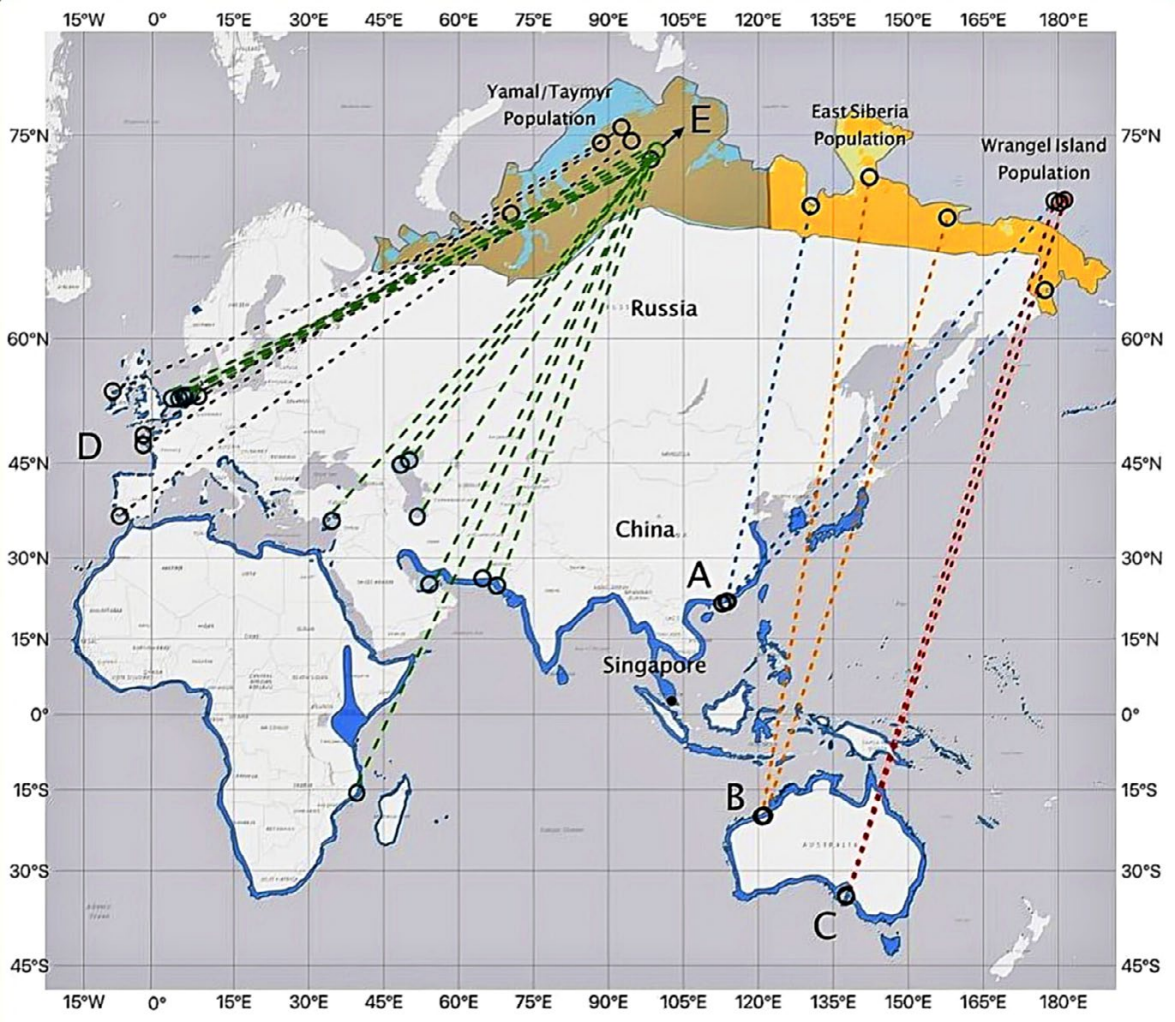
Breeding ground distributions of Yamal/Taymyr population (light blue over orange), East Siberia population (yellow over orange) and Wrangel Island populations (red) of Grey Plovers in the Palearctic as identified by Engelmoer and Roselaar (1998). Prior to our study 12 birds were tracked between the non‐breeding grounds and their breeding grounds in the Arctic; dashed lines on the map indicate the non‐breeding grounds of the bird and its breeding locations only and do not denote actual track lines. A—Birds tracked from Hong Kong in blue dashed lines (Croucher Foundation 2021), B—Northwest Australia in yellow dashed lines (Leung et al. 2016), C—South Australia in red dashed lines (Flaherty 2016), D—Europe (Exo, Hillig, and Bairlein 2019). Thirteen birds were tracked from breeding grounds in Taymyr Peninsula to non‐breeding grounds in Southern Europe, Africa and West Asia (Soloviev et al. 2023), represented by E in green dashed lines. Base map from Esri with Mercator projection. Species distribution map provided by BirdLife International (orange—breeding range, blue—non‐breeding range).

One such species is the Grey Plover ( *Pluvialis squatarola* ), which is the largest plover species that breeds on the Arctic Tundra. Unlike other plover species, it has an almost circumpolar arctic breeding distribution that ranges from the eastern White Sea, Russia east to Baffin Island, Canada (Byrkjedal and Thompson 1998; Poole et al. 2020). Using bird morphometrics, it was suggested that three populations of Grey Plover occur across its breeding range in the Palearctic (Engelmoer and Roselaar 1998; Tomkovich and Serra 1999; Exo and Stepanova 2001; Lappo, Tomkovich, and Syroechkovskly 2012). From west to east, they included the Yamal/Taymyr, East Siberia/Alaska and Wrangel populations (Figure 1). However, these differences are not absolute, as there is still considerable overlap in morphometric measurements between populations (Engelmoer and Roselaar 1998; Exo and Stepanova 2001).

While the occurrence of Grey Plovers along all the major global flyways is established, the exact routes and connections between wintering and breeding areas along these flyways have only been documented for Grey Plovers breeding in the Yamal and Taymyr Peninsular, Russia (Exo, Hillig, and Bairlein 2019; Soloviev et al. 2023) and in Arctic Alaska and Canada (Harrison and others, unpublished data). Minton and Serra (2001), using morphological measurements, suggested Grey Plovers breeding east of the Lena River in the northeastern Russian Arctic (i.e., East Siberia breeding population) winter in northwestern Australia, while plovers breeding in Wrangel Island, Russia were thought to winter in southeastern Australia. This was shown in 2016 when two Grey Plovers were tracked with satellite transmitters from their wintering grounds at Thompson Beach in the Gulf St Vincent, South Australia to their breeding grounds at Wrangel Island (Flaherty 2016), while two other birds were tracked from Northwest Australia to their breeding grounds in East Siberia (Leung et al. 2016, Figure 1). All four birds used portions of the Yellow Sea during north migration to reach their breeding grounds. In addition, 18 birds tracked from the Central Taymyr Peninsular migrated via the East Atlantic Flyway, Black Sea/Mediterranean Flyway, and West Asian/East African Flyway to Europe and Africa (Soloviev et al. 2023). Furthermore, three birds equipped with satellite transmitters at a wintering area in Hong Kong between November 2018 and January 2019 travelled north via the EAAF to their breeding grounds in Yakutia (=Sakha), Wrangel Island and Chukotka, respectively (Croucher Foundation 2021). However, none of the reports on satellite‐equipped birds from Australia and Hong Kong indicate plovers travel across Peninsular Malaysia, Singapore and Indo‐China.

The breeding location of Grey Plovers that winter on the Peninsular Malaysia and Singapore is currently unknown (Bamford et al. 2008; Li et al. 2009; Mundkur, Langendoen, and Watkins 2017). Up to 1200 Grey Plovers have been counted in Pulau Tengah, Selangor, Malaysia, a number that could account for more than 90% of the birds wintering in Peninsular Malaysia (Wells 1999). Previous records indicate that no more than 60 birds winter in Singapore (Wang and Hails 2007; Lim and Lim 2009; Wells 1999), with recent counts up to 85 wintering regularly at Chek Jawa Wetlands on Pulau Ubin (NParks, unpublished). Although there were seven plovers marked in Singapore between 2008 and 2019, no confirmed recoveries have occurred outside of Singapore. Migration patterns inferred from Australian‐tracked birds suggest that the species migrates along the EAAF to breeding areas in northeast Russia. However, satellite‐tracked Whimbrels ( *Numenius phaeopus* ) wintering in Singapore were demonstrated to use both the Central Asian Flyway (CAF) and the EAAF, with some birds crossing the Himalayas to reach their breeding grounds in north central Siberia (Li et al. 2020). Thus, it is unclear exactly where Grey Plovers wintering in Singapore will travel to reach their breeding grounds.

In this study, we used satellite tracking to investigate whether Grey Plovers wintering in Singapore are part of the East Siberia/Alaska or Yamal/Taymyr populations and how they migrated north and south between Singapore and their wintering area (Figure 1). We did not think the Singapore plovers were part of the Wrangel Island population because prior information suggested these birds wintered in south Australia. The westerly location of Singapore (relative to the EAAF) and the prior tracking information on Whimbrels and Common Redshanks ( *Tringa totanus* ) made it difficult to rule out their use of either the EAAF or CAF. We also investigated if the ground speed and flight path of the Grey Plover were supported by prevailing winds.

## 2 Methods

### 2.1 Capture and Satellite Tracking

Two Grey Plovers, both after‐hatch year birds, were caught in mist nets at night at Chek Jawa Wetlands (CJ, 1.41 ° N, 103.99° E) in Singapore from October to November 2017. Both birds were banded with a metal ring on the left tarsus and two plastic leg flags (green/white) on the right tibia and morphometrics (wing, bill to skull, bill to head, tail, tarsus minimum and body mass) were recorded. They were also equipped with 5‐g solar‐powered satellite transmitters (Platform Transmitter Terminals—PTT, Microwave Telemetry Inc., USA) using leg‐loop harnesses (Rappole and Tipton 1991). Satellite transmitters and their harnesses were < 3% of the body weight as suggested by Phillips, Xavier, and Croxall (2003) to minimise impact on their migration. Data were successfully obtained for a full migration cycle from one Grey Plover (identified as B3 by its engraved flag number) while transmissions from the second bird were lost in February 2018 before the northward migration.

The transmitters were programmed to operate continually with a duty cycle of 10 h on and 48 h off. This program ensured batteries were continually charged for long‐term monitoring but also allowed data transmission when the transmitters were fully charged. Bird locations and times derived from the transmitters were obtained from Collecte Localisation Satellites through the Argos website (www.argos‐system.org). We used Class 1 (accuracy between 500 and 1500 m), Class 2 (accuracy between 250 and 500 m) and Class 3 (accuracy within 250 m) data for analysis and mapping. However, we also used less accurate locations (i.e., Classes 0, A, B) when the birds were moving to allow us to calculate departure and arrival dates, flight path during migration, and to analyse a bird's speed in relation to the wind.

### 2.2 Stopover Sites

Given the PTTs' operating schedule, we were unable to identify stopovers that lasted < 2 days when the tracker was off for charging. We identified stopover sites by combining the locations together when they were within the approximate error range of 150 km for > 2 days, preceded and followed by directional movements > 150 km (Li et al. 2020).

### 2.3 Migration Distances, Ground Speeds and Direction

Ground speeds were calculated by dividing the distance travelled by the time expended between consecutive stopover locations and assumed to be constant. It was not possible to calculate the true distance flown by our single tagged bird due to the PTTs' operating schedule. Instead, we calculated the great circle distance between all consecutive data points as it travelled between their non‐breeding and breeding grounds. We considered this the minimum flight distance travelled. Ground speed and direction of travel were calculated using the ‘move’ package in R (Kranstauber, Smolla, and Scharf 2020). Also due to the PTTs' operating schedule, we excluded locations where apparent speeds were < 20 km h^−1^ as this could include undetected short stopovers (Li et al. 2020). This amounted to 49 locations for analyses of the ground speed of Grey Plover B3. This can be separated into three periods: northward migration from Singapore to the breeding site (*n* = 24), post‐breeding (breeding site to point the bird began migrating south) (*n* = 3), and southward migration from the end of the post‐breeding period to Singapore (*n* = 22).

### 2.4 Wind Data

To gain a better understanding of how winds affected plover migration, we used wind speed and direction from ECMWF's ERA5 reanalysis dataset (Hersbach et al. 2020) for the three migration periods. Wind speed and direction at surface (PLsurface: 10 m above ground), 1000 mbar (PL1000: ~100 m asl), 850 mbar (PL850: ~1500 m asl), 700 mbar (PL700: ~3000 m asl) and 500 mbar (PL500: ~5500 m asl) were obtained using the Env‐DATA annotation tool in Movebank (Dodge et al. 2013). Since only one study has reported the flight height of Grey Plovers (mean flight height: 1726 ± 685 m asl (Green 2004)), we used a range of altitudes instead as it likely covered all flight paths based on flights of other shorebirds tracked from Singapore (Li et al. 2020). Additionally, we calculated wind support based on formula by Safi et al. (2013): *v*_w_*cos(*α*), where *v*_w_ is wind speed and *α* is the difference between the bird's track direction and wind direction. To evaluate the effect of wind support on the ground speeds of the plover, we used linear models with ground speed as the response variable and wind support at each of the altitudinal levels as the explanatory variable. This analysis was conducted separately for the northward and southward migration periods, but not for post‐breeding period due to small sample size.

In addition to numerical computations for wind variables, regional wind conditions were also visualised using the ERA5 reanalysis dataset for latitudes 12° N to 52° N and longitudes 81° E to 116° E. R packages ‘ncdf4’ (Pierce 2019), and ‘tidync’ (Sumner 2020) were used to import and extract wind data from the downloaded NetCDF file. The package ‘ggquiver’ (O'Hara‐Wild 2019) was used to produce quiver plots that show the regional wind conditions as vectors. We conducted this analysis for 20152020 to evaluate whether consistent winds occurred in the area of interest by comparing if the mean and standard deviation of wind support at each pressure level overlap among the years.

To better understand the effect winds might have on the eastward migration of the bird when north of the Himalayan Mountains, we interpolated additional locations along the migration path using the ‘move’ package in R (Kranstauber, Smolla, and Scharf 2020). This was to increase the number of locations that could be associated with the wind along the migration path and assumed that ground speed and direction were constant between adjacent locations. All analyses were conducted using R (version 2023.03.0; R Core Team 2023).

## 3 Results

### 3.1 Morphometrics

Based on the morphometrics of both Grey Plovers (Table S1), it is impossible to determine their breeding population location with confidence as the morphometrics that were measured overlapped with those in various populations (Engelmoer and Roselaar 1998).

### 3.2 Migration Route, Timing and Stopover Sites

**FIGURE 2:**
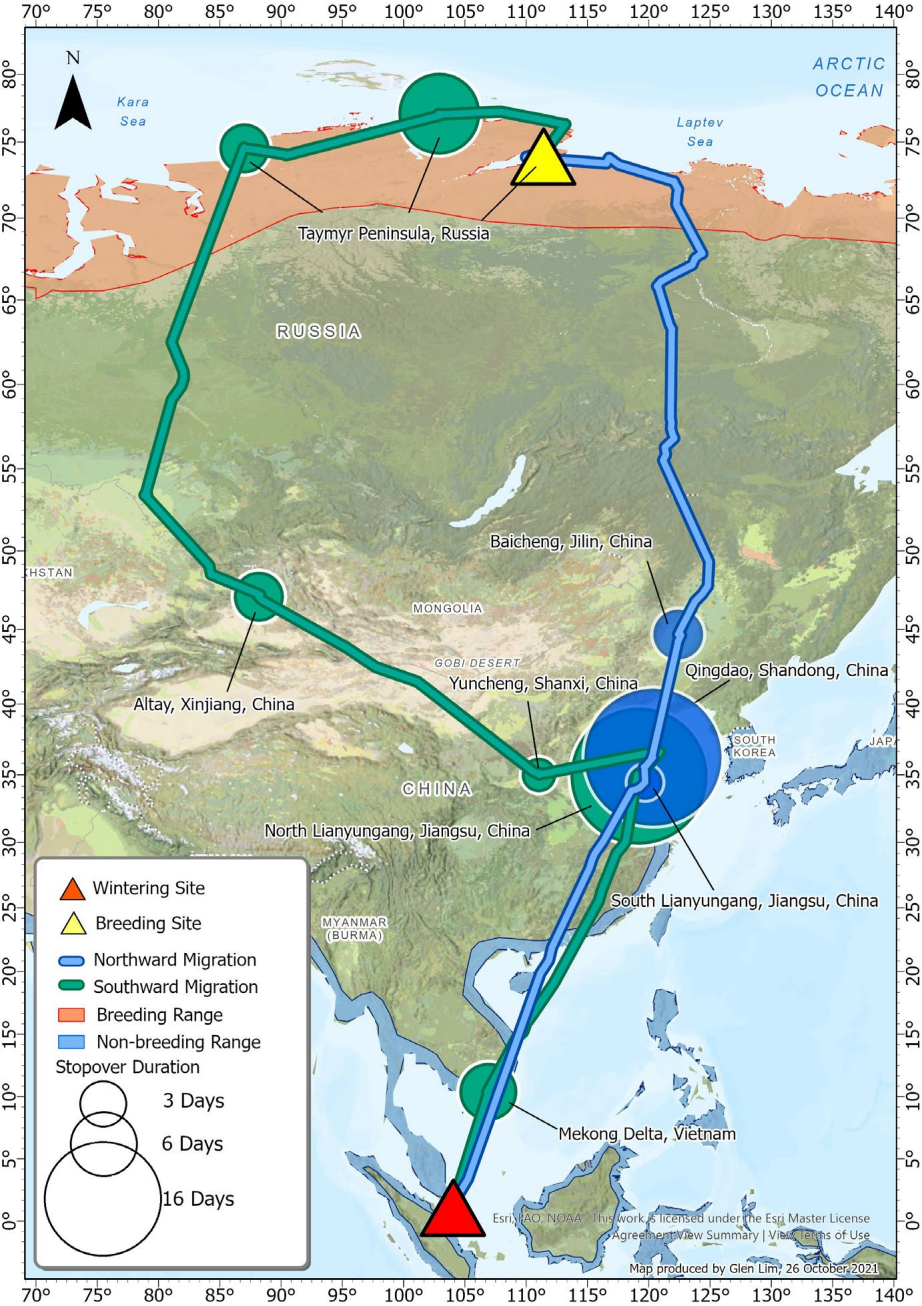
Migration route and stopover sites of Grey Plover B3 based on satellite tracking in 2018. See Table 1 for details of each stopover location. Combination of World topographic map (Esri 2012) and National Geographic World map (Esri 2011) with Mercator projection was used to produce the map. Species distribution maps were provided by BirdLife International. Breeding range of Yamal/Taymyr population is west of ~120° E and the East Siberia/Alaska population is east of ~120° E (Engelmoer and Roselaar 1998).

The Grey Plover B3 spent 46 days travelling 8645 km on its northward migration along the EAAF from Singapore to Bolshoy Begichev Island, east of Taymyr Peninsula in north‐central Siberia, Russia. Along the route, it stopped thrice at sites in northeast China, including coastal areas 40 km south of Lianyungang in Jiangsu Province, 20 km north from Qingdao in Shandong Province, and an inland wetland south of Baicheng in Jilin Province (Table 1, Figure 2), with the longest stopover being 16 days. Then, it stayed for a duration of 50 days in Siberia, which exceeds the timeframe for a successful breeding attempt (approximately 46 days) (Höhn 1957; Hussell and Page 1976). Hence, we termed this location as its breeding location.

**TABLE 1:** Migration timing and stopovers of Grey Plover B3 based on satellite tracking in 2018.

Arrival date	Departure date	Migration status	Location	Duration (days)	Lat (°N)	Long (°E)
	29 April 2018	Departed	Chek Jawa, Singapore	—	1.40	103.99
4 May 2018	6 May 2018	NW Stopover	South Lianyungang, Jiangsu, China	3	34.45	119.70
11 May 2018	27 May 2018	NW Stopover	Qingdao, Shandong, China	16	36.26	120.31
29 May 2018	3 June 2018	NW Stopover	Baicheng, Jilin, China	5	44.68	122.39
14 June 2018	3 August 2018	Breeding	Bolshoy Begichev Island, East of Taymyr Peninsula, north‐central Siberia, Russia	50	74.31	111.53
8 August 2018	17 August 2018	SW Stopover	North‐central Taymyr Peninsula, Krasnoyarsk Kari, Russia	9	77.00	102.90
19 August 2018	24 August 2018	SW Stopover	Northwestern Taymyr Peninsula, Krasnoyarsk Kari, Russia	5	74.57	87.01
31 August 2018	5 September 2018	SW Stopover	Altay, Xinjiang, China	5	47.11	88.16
6 September 2018	9 September 2018	SW Stopover	Yuncheng, Shanxi, China	3	35.00	111.03
19 September 2018	5 October 2018	SW Stopover	North Lianyungang, Jiangsu, China	16	34.89	119.19
8 October 2018	14 October 2018	SW Stopover	Mekong Delta, Vietnam	6	10.39	106.93
17 October 2018		Arrived	Chek Jawa, Singapore	—	1.39	104.02

*Note:* Sites where birds were determined to stop for at least 2 days are listed as locations and depicted in Figure 2.

After breeding, Grey Plover B3 spent 75 days travelling 12,103 km to return to its wintering grounds in Singapore (Table 1, Figure 2). Part of its return migration involved flying westwards to the north‐central and northwestern Taymyr Peninsula where the bird staged for 9 and 5 days respectively. From here, the bird moved south along the CAF to Altay, Xinjiang Autonomous Region, then migrated eastward to Yuncheng in Shanxi Province. Then, it migrated south along the EAAF, with stopovers at Yuncheng in Shanxi Province, Lianyungang in Jiangsu province in China, and on the Mekong Delta in Vietnam, before reaching Singapore (Figure 2).

### 3.3 Wind Support During Migration

**FIGURE 3:**
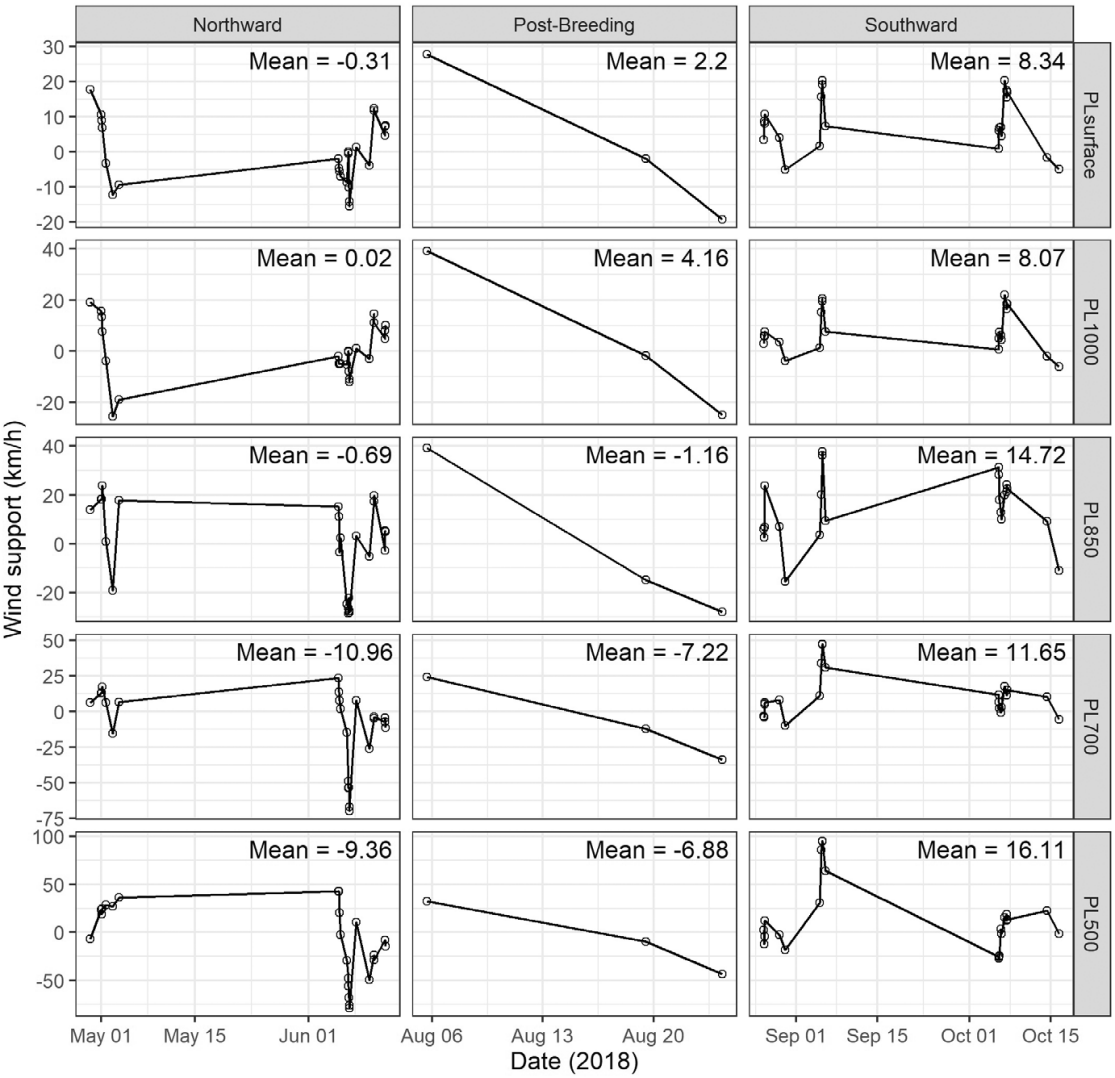
Distribution and mean wind support at various pressure levels for the north, post‐breeding, and south migration path of the Grey Plover B3 in 2018. See text for definitions of these three time periods. Positive values indicate wind support while negative values indicate headwinds.

Wind support had a positive effect on Grey Plover B3's ground speed at all pressure levels in both northward and southward migration but was overall more favourable during the southward migration (Table 2, Figure 3). In the northward migration period, this relationship was statistically significant for pressure levels above 700 mbar, with wind support accounting between 17% and 24% variability observed in ground speed (Table 2). In the southward migration period, this relationship was statistically significant for all pressure levels except 850 mbar, with wind support accounting between 18% and 24% variability observed in ground speed (Table 2). Our analysis of wind conditions suggests that the westward migration by the B3 plover post‐breeding season is unlikely to do with the wind enabling their migratory flights, as the wind conditions were not consistently favourable (Figure 3).

**TABLE 2:** Summary of the coefficients of the linear models investigating the effect of wind support of Grey Plover B3 ground speed at various pressure levels for northward and southward migration periods.

Pressure level	Northward migration (*N* = 24)				Southward migration (*N* = 23)			
	Coefficient	SE	*p*	*R* ^2^	Coefficient	SE	*p*	*R* ^2^
PLsurface	0.74	0.33	**0.03**	0.19	1.55	0.67	**0.03**	0.20
PL1000	0.69	0.26	**0.02**	0.24	1.32	0.61	**0.04**	0.18
PL850	0.36	0.17	**0.04**	0.17	0.74	0.42	0.09	0.13
PL700	0.18	0.11	0.13	0.10	0.91	0.35	**0.02**	0.24
PL500	0.09	0.09	0.29	0.05	0.42	0.16	**0.02**	0.24

*Note:* Statistically significant effects (*p* < 0.05) are indicated in bold. Interpolated locations were not included in the analysis.

**FIGURE 4:**
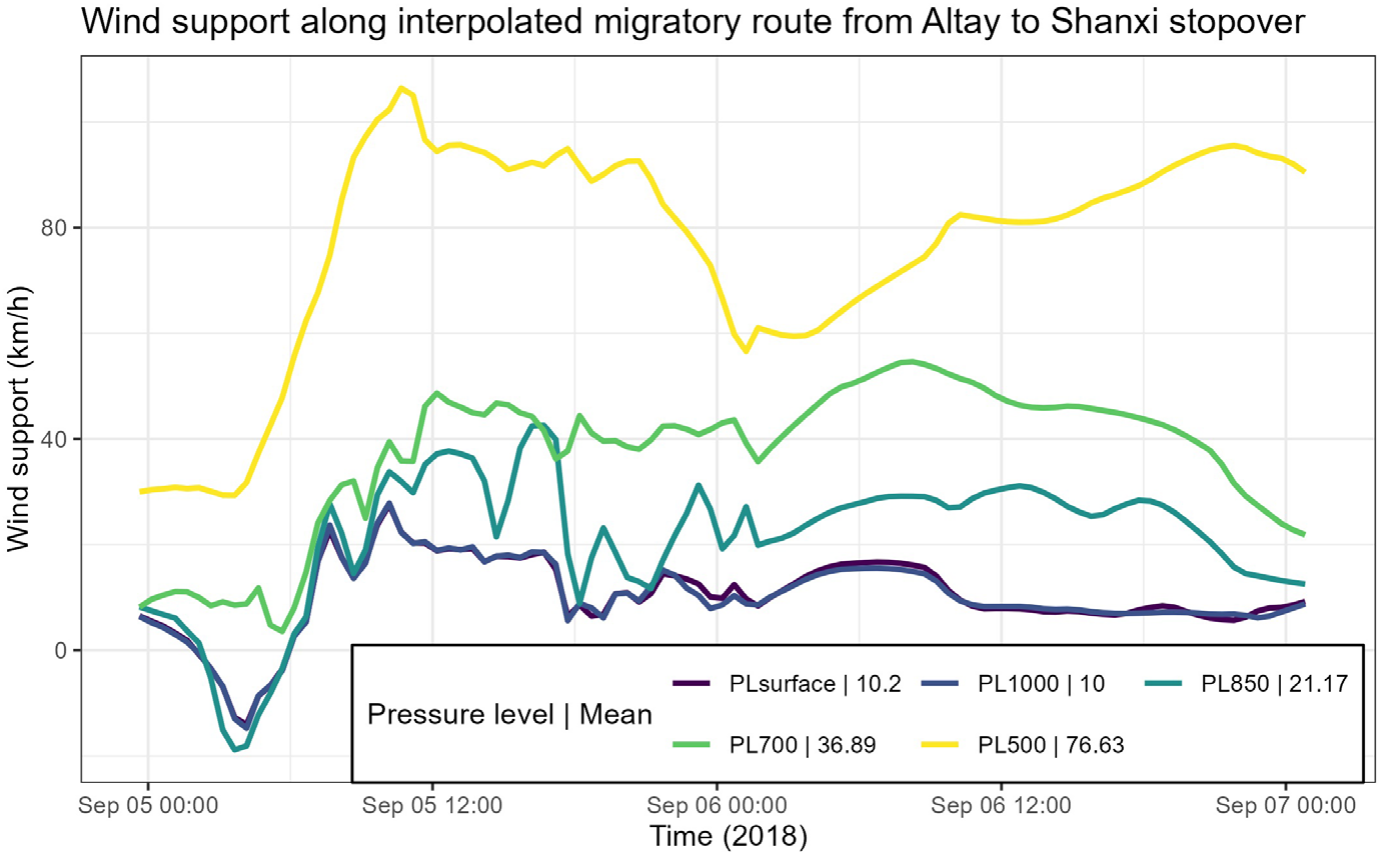
Wind support at various pressure levels for the migratory path of Grey Plover B3 between Altay in the Xinjiang Autonomous Region and Yuncheng in Shanxi Province, China. Interpolated movement data were used to produce the Figure.

During southward migration, the plover took a non‐stop southeastward flight from Altay, Xinjiang Autonomous Region to Yuncheng, Shanxi Province before reaching the east China coast (Figure 2). During this eastward movement, the bird's speed was aided by strong wind support (Figure 4), allowing it to travel at a ground speed of 93.4 km/h for 2260 km. Wind support at various pressure levels from Altay to Yuncheng generally followed a similar trend between 2015 and 2020 (Table S2, Figure S1). In addition, most of the wind support throughout the migration path was positive, that is, tailwind (Figure 4).

## 4 Discussion

### 4.1 Breeding Grounds and Geographic Populations of the Grey Plover

Engelmoer and Roselaar (1998) suggested three breeding populations of Grey Plovers exist across the Palearctic based on morphometrics (Figure 1). However, only the Wrangel population of plovers has been recognised as a separate subspecies (*P.s. tomkovichi*); the other two populations include those breeding in the Yamal/Taymyr and East Siberia/Alaska. Differences in morphometrics of birds from these two regions are minimal, making the assignment of breeding location using morphometrics unreliable (Engelmoer and Roselaar 1998). The border between these two populations is suspected to be somewhere between the Taymyr and East Siberia. Our finding that this single Grey Plover B3, which wintered in Singapore, bred in the north‐central Siberia just east of the Taymyr Peninsula is notable, because plovers breeding in this area are thought to migrate south via the East Atlantic Flyway and winter in Western Europe (Exo and Wahls 1996; Tomkovich, Lappo, and Syroechkovski Jr. 2000; Soloviev et al. 2012; Exo, Hillig, and Bairlein 2019). This suggests several possibilities. First, the Singapore Grey Plover is part of the Yamal/Taymyr population and plovers from this population migrate down multiple flyways. Second, the border between the two plover populations is further to the west and B3 belongs to the East Siberia/Alaska population. Or third, there exists a third population of Grey Plovers that breeds between the Yamal/Taymyr and East Siberia/Alaska populations and winters in Central and Southeast Asia. Focused studies, including more extensive migration tracking and DNA analyses, are needed to evaluate these alternatives. Additional research is also needed to refine the migratory connectivity of plovers wintering in Hong Kong as tagged birds from this area have been shown to breed in Yakutia (=Sakha), on Wrangel Island, and Chukotka (Croucher Foundation 2021).

### 4.2 Post‐Breeding Movement

The migration of the single Grey Plover westward to the northern Taymyr Peninsula after breeding was unexpected, although several anecdotal observations of plovers and other species suggest it may not be unusual. First, a Grey Plover tagged in South Australia and later documented breeding on Wrangel Island underwent a west to east pre‐breeding migration from northern Yakutia to Wrangel Island and an east to west post‐breeding migration from Wrangel Island to New Siberian Islands before heading southward (Flaherty 2016). Second, one of the Grey Plovers tagged in Hong Kong also undertook a northwest migration to the Taymyr Peninsula after breeding in northern Siberia (Croucher Foundation 2021, K. Leung, personal communication). Third, observations in the north‐central Taymyr Peninsula indicate the number of adult Grey Plovers increases significantly in the second half of summer, as birds are observed flying predominantly west and northwest through this area at this time (Tomkovich, Soloviev, and Syroechkovski Jr. 1994). Additionally, northward migration after breeding has been documented for some Siberian populations of the Bar‐tailed Godwit (Tomkovich, Soloviev, and Syroechkovski Jr. 1994; Battley et al. 2012; Bom et al. 2021).

The westward post‐breeding migration by our tracked Grey Plover did not appear to be related to winds supporting its movements but might be explained in several other ways. First, birds may need to accumulate sufficient reserves after breeding to support their southward flight over the expansive Russian boreal belt. Moving west might be beneficial if fuel‐deposition rates are better later in the summer in the Arctic Taymyr Peninsula, which is further north and phenologically delayed. Tulp and Schekkerman (2008) showed that arthropod biomass continued to increase until the middle of August in Northwest Taymyr Peninsula in some years. A study of Tipuloidea flies and their larvae in the Siberian Arctic has shown that this food resource is abundant in the arctic tundra subzone and most accessible for waders beginning in mid‐July (Lantsov and Chernov 1987), when hatching of chicks of Grey Plovers starts locally and not long before the influx of numbers of migrant Grey Plovers (Tomkovich, Soloviev, and Syroechkovski Jr. 1994).

Additionally, the westward migration of the Grey Plover may serve the purpose of investigating future breeding or staging sites, a behaviour advantageous for birds to increase its reproductive success (Reed et al. 1999; Wauchope et al. 2017). This could be due to changing environmental conditions, particularly driven by climate change. Studies suggest that post‐breeding movements, such as the westward migration observed, are influenced by shifts in the distribution of climatically suitable breeding conditions in the Arctic (Wauchope et al. 2017). For example, southern breeding species have expanded into the Russian Arctic during the 20th century, and there are reports of breeding range extensions in Arctic shorebirds like the Semipalmated Plover ( *Charadrius semipalmatus* ) and Semipalmated Sandpiper ( *Calidris pusilla* ) (Lappo, Tomkovich, and Syroechkovskly 2012; Meltofte 2013). Additionally, post‐breeding movements, such as those observed in the Common Redshank from Sichuan Province to Qinghai Province, reflect birds' efforts to acquire information on new breeding sites amidst changing environmental dynamics (Li et al. 2020).

### 4.3 West to East Migration to Avoid Himalayan Crossing

**FIGURE 5:**
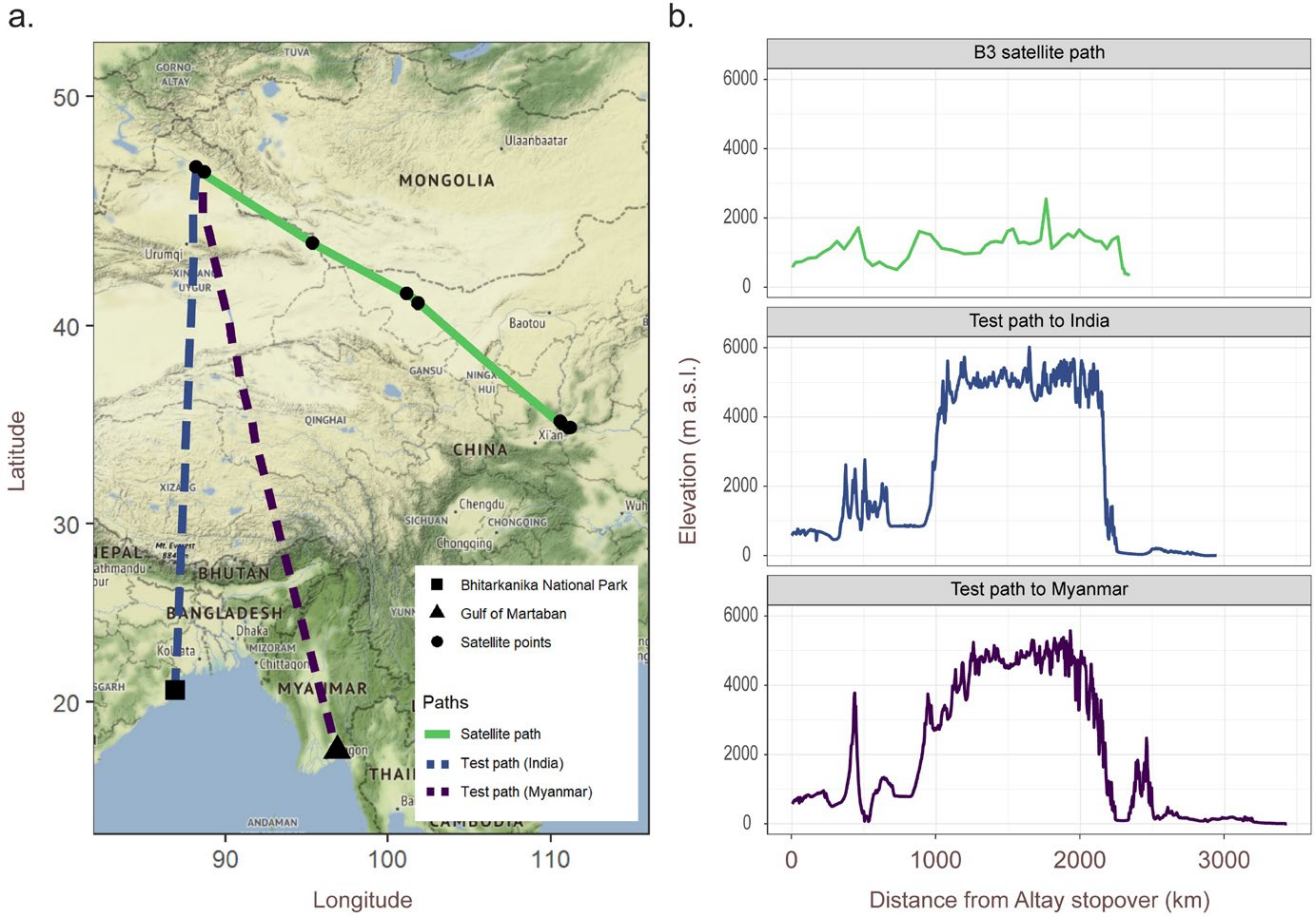
(a) Map and (b) topographic profile along the Grey Plover B3's migratory path from Altay in Xinjiang Autonomous Region to Yuncheng in Shanxi Province, and two other hypothetical southward migratory routes to Bhitarkanika National Park, India and to the Gulf of Martaban, Myanmar. The hypothetical routes were selected based on stopovers used by satellite‐tracked Whimbrel as described in Li et al. (2020) and are more direct route to Singapore.

Our Grey Plover's southward travel was vastly different than a Whimbrel tagged in Singapore in the same year. The Whimbrel travelled directly from Central Asia to Singapore by crossing the Himalayan mountains and stopping at India and Thailand (Li et al. 2020). In contrast, the Grey Plover B3 migrated eastward from the Xinjiang Autonomous Region to the east coast of China, and by doing so, took a much longer route (~2000 km further). The avoidance of the Himalayan mountains meant that the Grey Plover B3 could fly at a much lower altitude, not exceeding 2000 m asl as opposed to the 5000–6000 m asl required to cross the Himalayas (Figure 5). We also noted significant wind support for the Grey Plover B3 during a portion of the individual's southward migration as it flew from Altay to Yuncheng before reaching the east coast of China. An evaluation of wind conditions over the same area during the same migration period from 2015 to 2020 indicated winds were consistently favourable over the years at 500700 mbar (Figure S1). Further, wind support is also generally poorer if the bird had migrated across the Himalayas towards Bhitarkanika National Park in India or the Gulf of Martaban (=Mottama) in Myanmar (Figures S2 and S3)—two stopover sites used by a whimbrel tracked by Li et al. (2020) before and after crossing the Himalayas.

A west to east migration route in the north of the Himalayan mountains may be more commonly used by shorebirds during southward migration than previously known. Other migratory waterbirds, such as the Relict Gull ( *Larus relictus* ), which breeds in Hongjian Nur in northern China and winters on the east coast of China, have been documented using this migration route regularly (Liu et al. 2017). A Sanderling ( *Calidris alba* ) flagged in South Australia and later recorded in Xinjiang Autonomous Region in northwest China could have possibly used this route for migration as well (Deakin University 2021).

While there is no direct evidence that Grey Plovers regularly cross the Himalayan mountains during their southward migration, the most probable evidence to date is from a Grey Plover ringed in Sasykkol Lake, Kazakhstan in August 1989 that was later recovered in Point Calimere, Tamil Nadu in southeast India in January 1990 (Balachandran, Katti, and Manakadan 2018). However, there is no certainty that the bird migrated over the Himalayas. Individuals have been observed in the Tibetan‐Himalayan region at Upper Indus Valley (Delany et al. 2017) and Qinghai Lake in interior China (Lu 2018), suggestive of a Himalayan migration. Thus, it is possible that some birds migrate over the Himalayans and others take an easterly route like the B3 Grey plover.

## 5 Limitations

Considering that we had successfully tracked only one Grey Plover, it is uncertain if its migration path is an anomaly or typical among this species that winter in Singapore. Furthermore, since the satellite transmitter had downtime in between transmission periods, the knowledge of certain flight movements could have been incompletely recorded.

## 6 Conclusion

Overall, the tracking of this individual Grey Plover has prompted inquiries into the demarcation of breeding populations and the potential existence of a novel population. Our study also provided additional support that shorebirds make longitudinal migrations in the Arctic to possibly to acquire sufficient fat reserves for a successful southward migration or for prospecting future breeding/staging sites. Additionally, our findings offer supplementary evidence indicating the occurrence of longitudinal migrations north of the Himalayas, where birds may capitalise on the prevailing easterly winds for their migration. Further study is required to understand whether B3's route is typical or unusual among Grey Plovers breeding in the Russian Arctic and wintering in South and Southeast Asia.

## Author Contributions

**David Li:** conceptualization (equal), data curation (lead), formal analysis (lead), investigation (lead), methodology (equal), project administration (equal), writing – original draft (lead), writing – review and editing (equal). **Max De Yuan Khoo:** formal analysis (equal), methodology (equal), software (equal), visualization (equal), writing – review and editing (lead). **Richard B. Lanctot:** conceptualization (equal), writing – original draft (equal), writing – review and editing (equal). **Pavel S. Tomkovich:** conceptualization (equal), writing – original draft (equal), writing – review and editing (equal). **Zhijun Ma:** conceptualization (equal), writing – original draft (equal), writing – review and editing (equal). **Jun Rui Chow:** formal analysis (equal), software (equal), visualization (equal). **Malcolm Chu Keong Soh:** writing – review and editing (equal). **Shufen Yang:** conceptualization (equal), methodology (equal), project administration (lead), writing – review and editing (equal). **Choon Beng How:** conceptualization (equal), methodology (equal), project administration (lead), writing – review and editing (equal). **Adrian Loo:** resources (equal), supervision (lead), writing – review and editing (equal). **Robert Teo:** conceptualization (equal), methodology (equal), project administration (lead), writing – review and editing (equal). **Liang Jim Lim:** resources (equal), supervision (lead), writing – review and editing (equal). **Chee Chiew Leong:** funding acquisition (lead), resources (lead), writing – review and editing (equal). **Kenneth Boon Hwee Er:** conceptualization (lead), funding acquisition (lead), methodology (equal), resources (lead), writing – original draft (equal), writing – review and editing (equal).

## Ethics Statement

Research protocols were approved by National Parks Board, Singapore. All field work was conducted in accordance with regulations of the Parks and Trees Act and the Wild Animals and Birds Act.

## Conflicts of Interest

The authors declare no conflicts of interest.

## Supporting information


Data S1.

## Data Availability

The bird tracking dataset that support the findings of this study are openly available in a public repository (Dryad) at DOI: 10.5061/dryad.8931zcs1m.
