# Supplementary material for: Migration of Wintering Grey Plover From Southeast Asia to North‐Central Siberia Challenges Breeding Population Delineations in Russia
Source: Ecol Evol. 2025 Feb 24;15(2):e70815. doi: 10.1002/ece3.70815 (PMC11850438; doi:10.1002/ece3.70815)
Supplement: Supplementary file 1 — Data S1. [file ECE3-15-e70815-s001.docx]

Table S1. Morphometrics of the two Grey Plovers that were caught at Chek Jawa Wetlands in Singapore.

| **Identity** | **Date** | **Wing length (mm)** | **Bill to skull length (mm)** | **Bill to head length (mm)** | **Tail length (mm)** | **Tarsus minimum length (mm)** | **Body mass (g)** |
| --- | --- | --- | --- | --- | --- | --- | --- |
| B3 | 12-Oct-17 | 199 | 38.5 | 68.4 | 80 | 45 | 260 |
| B5 | 9-Nov-17 | 208 | 39 | 71.2 | 74 | 48 | 235 |

Table S2. Mean and standard deviation (SD) of wind support at various pressure levels from 2015 - 2020 for the migratory path between Altay in the Xinjiang Autonomous Region and Yuncheng in Shanxi Province, China used by the Grey Plover B3.

|  | **PL500** | | **PL750** | | **PL850** | | **PL1000** | | **Plsurface** | |
| --- | --- | --- | --- | --- | --- | --- | --- | --- | --- | --- |
| **Year** | **Mean** | **SD** | **Mean** | **SD** | **Mean** | **SD** | **Mean** | **SD** | **Mean** | **SD** |
| 2015 | 40.40 | 11.74 | 23.05 | 10.61 | -0.23 | 12.09 | -1.20 | 7.74 | -1.50 | 8.08 |
| 2016 | 39.51 | 17.97 | 17.48 | 13.42 | 10.54 | 8.45 | 4.25 | 6.27 | 4.25 | 6.22 |
| 2017 | 32.59 | 15.03 | 7.53 | 7.08 | 7.08 | 7.59 | -0.18 | 5.35 | 0.07 | 5.23 |
| 2018 | 76.63 | 20.83 | 36.89 | 13.82 | 21.17 | 12.70 | 10.00 | 7.41 | 10.20 | 7.41 |
| 2019 | 15.11 | 10.87 | 4.15 | 10.76 | -8.16 | 14.53 | -4.11 | 9.79 | -4.28 | 9.83 |
| 2020 | 41.61 | 8.85 | 24.87 | 14.33 | 13.97 | 16.64 | 7.95 | 12.82 | 7.92 | 12.78 |


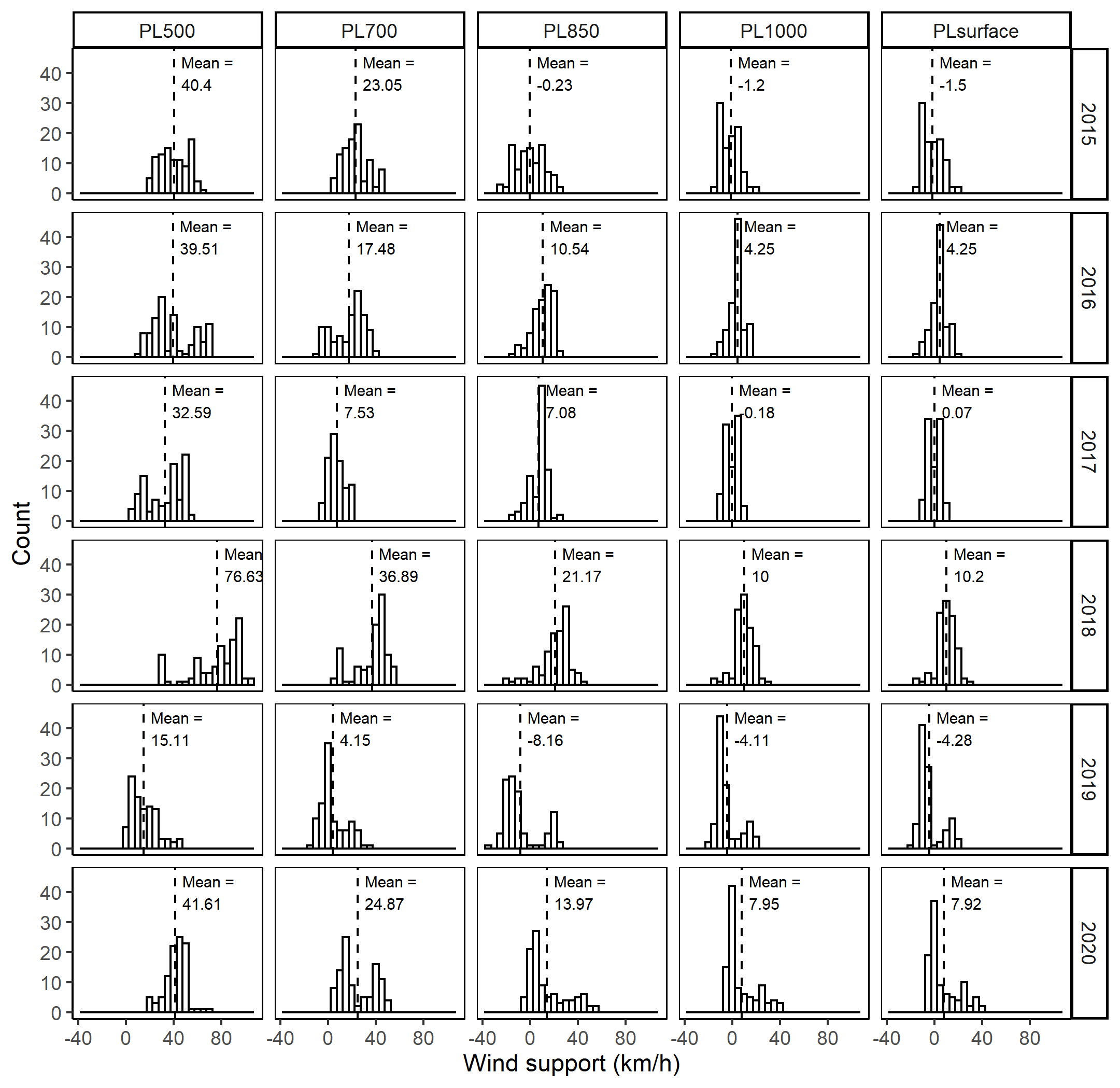
 Figure S1. Distribution and mean wind support at various pressure levels from 2015 - 2020 for the migratory path between Altay in the Xinjiang Autonomous Region and Yuncheng in Shanxi Province, China used by the Grey Plover B3. Count refers to the number of data points in each bin of wind support (bin width = 5). Interpolated migration data were used to produce the Figure.


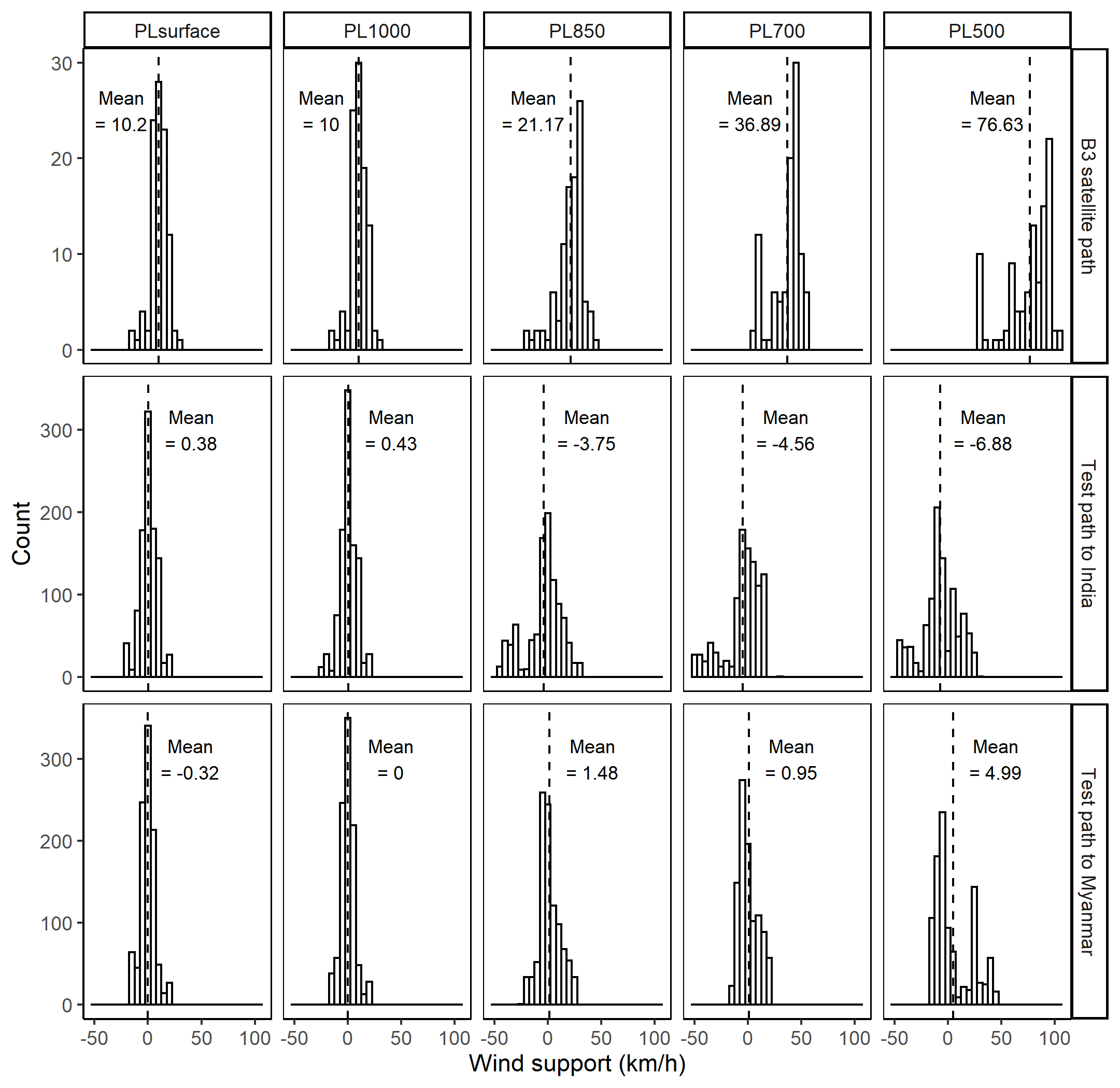
 Figure S2. Distribution and mean (vertical dashed line) wind support at various pressure levels along the Grey Plover B3’s migratory path if it were to migrate from Altay in Xinjiang Autonomous Region to Yuncheng in Shanxi Province, China; Bhitarkanika National Park, India; or Gulf of Martaban, Myanmar in 2018. Count refers to the number of data points in each bin of wind support (bin width = 5). Interpolated migration data were used to produce the Figure.


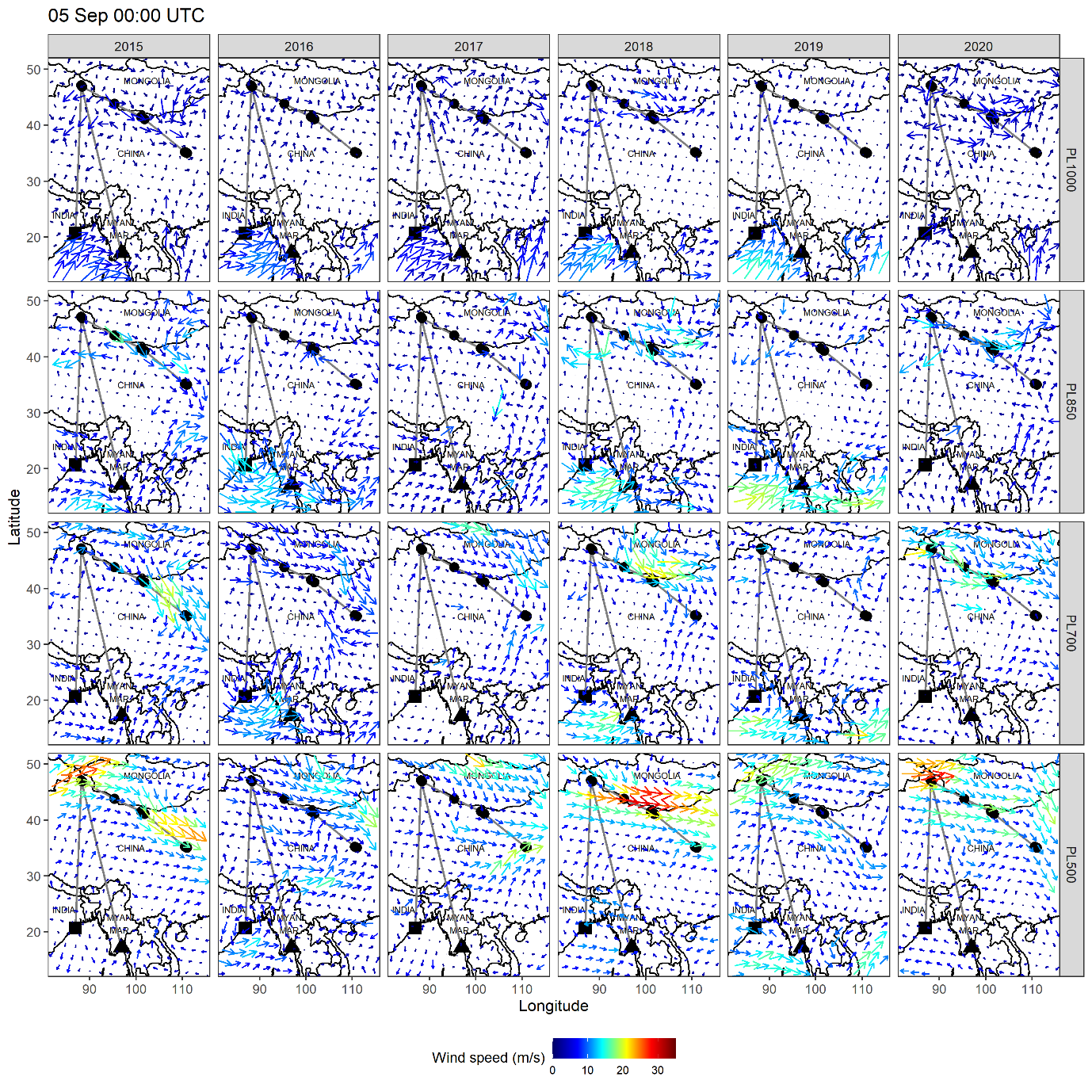
 Figure S3. Quiver plot showing wind direction and speed at the various pressure levels on the 5 September (00:00 hr) between 2015 and 2020. This date is in the middle of Grey Plover B3’s southward migration. Black circles represent the satellite-tracked points from the Altay stopover to the Yuncheng stopover; the black square represents the hypothetical Bhitarkanika National Park stopover site; and the black triangle represents the hypothetical Gulf of Martaban stopover site.
